# The Impact of Hurricane Katrina on Students’ Behavioral Disorder: A Difference-in-Difference Analysis

**DOI:** 10.3390/ijerph120505540

**Published:** 2015-05-22

**Authors:** Xian-Liang Tian, Xian Guan

**Affiliations:** 1 Center for Industrial and Business Organization, Dongbei University of Finance and Economics, No. 217 Jianshan Street, Shahekou District, Dalian 116025, Liaoning Province, China; 2 Center for Social Work Development and Research, Southwestern University of Finance and Economics, No. 55 Guanghuacun Rd., Chengdu 610072, Sichuan Province, China; E-Mail: guanxian@swufe.edu.cn

**Keywords:** hurricane, Katrina, discipline, difference-in-difference, behavior

## Abstract

*Objective*: The objective of this paper is to examine the impact of Hurricane Katrina on displaced students’ behavioral disorder. *Methods*: First, we determine displaced students’ likelihood of discipline infraction each year relative to non-evacuees using all K12 student records of the U.S. state of Louisiana during the period of 2000–2008. Second, we investigate the impact of hurricane on evacuee students’ in-school behavior in a difference-in-difference framework. The quasi-experimental nature of the hurricane makes this framework appropriate with the advantage that the problem of endogeneity is of least concern and the causal effect of interest can be reasonably identified. *Results*: Preliminary analysis demonstrates a sharp increase in displaced students’ relative likelihood of discipline infraction around 2005 when the hurricane occurred. Further, formal difference-in-difference analysis confirms the results. To be specific, post Katrina, displaced students’ relative likelihood of any discipline infraction has increased by 7.3% whereas the increase in the relative likelihood for status offense, offense against person, offense against property and serious crime is 4%, 1.5%, 3.8% and 2.1%, respectively. *Conclusion*: When disasters occur, as was the case with Hurricane Katrina, in addition to assistance for adult evacuees, governments, in cooperation with schools, should also provide aid and assistance to displaced children to support their mental health and in-school behavior.

## 1. Introduction

Hurricane Katrina was the one of most devastating natural disasters in the United States that hit Southeast Louisiana in August 2005. Around 2500 people died in the hurricane and property damage was approximately $108 billion [1]. The affected population of Katrina faced many socioeconomic problems. For example, eighty percent of New Orleans was flooded and many homes were destroyed. Approximately one million people migrated to different places as 35,000 refugees moved to Texas, 24,000 to Alabama and 15,000 went to Northern Louisiana [2]. The lives of Katrina migrants were not good as they did not find many job opportunities. This situation persisted more than a year [3,4]. This disaster also brought forward psychosocial distress [5,6] as one third of the affected adults faced psychological problems [7,8]. It was found that the violence and suicide completion rates were three and 14 times higher, respectively than the baseline rates in the U.S. [9].

Other consequence of the hurricane was dislocation of nearly 180,000 public school students, consisting of around 25% of Louisiana public school enrollment on that year [2]. Some of them moved to other states in the U.S (e.g., 45,000 dislocated students went to Texas, 8000 to Georgia, 5500 to Florida, 5000 to Mississippi, *etc*.) [10]. Ninety-three percent of the evacuee students were from the most affected parishes (Orleans, Jefferson, Plaquemines and St. Bernard), but only 69% of them stayed in those parishes next spring as the result of relocation [11]. Many studies investigated the consequent psychological problems of displaced students. One report showed that evacuee students had long-term (two years and more) psychological problems [12]. Another study found that displaced students were more likely to experience general psychological distress and posttraumatic stress [13]. These symptoms happened with higher probabilities among those being more disrupted [14] and tended to be worsening over time [15]. It was also observed that displaced students were more prone to have negative emotions [16].

A natural expectation is that the psychological disorders caused by the hurricane could lead to behavioral problems among affected students. However, literature discussing behavioral change pre and post the hurricane of those students are rare. This paper investigates the change of displaced students’ in-school discipline records after the hurricane in a difference-in-difference (DID) framework using Louisiana Department of Education administrative data. There are many advantages of the DID approach and one is that it yields well-founded causality from treatment to outcome when the treatment is exogenous [17]. At this point, two related research papers are worth mentioning. One is similar to our paper in that the authors studied Katrina in a DID framework but it looked into the effect of the hurricane on students’ academic performance while this paper investigates the change of in-school behavior [11]. The other one studied the change of students’ discipline post Katrina as this paper does. However, the previous paper mainly examined the effect of influx of evacuees on native students’ discipline in order to study peer effects whereas our paper looks into the change of behavior of student evacuees [10].

### Related Theories and Hypothesis

Two theories in the literature shed light on the effects of Hurricane Katrina on displaced students’ in-school behavior. One is Robert Agnew’s general strain theory (GST) in criminology, which has been one of the most popular theories to explain reasons behind crime [18,19,20]. The basic idea of Agnew’s theory is quite straightforward: confronted with strain, people may commit delinquency to release their pressure [21]. Since GST was put forward, a lot of research has been dedicated to provide it empirical supports in the framework of multivariate regression [22,23,24,25,26,27,28]. Agnew posits that strain generally comes from three sources. These sources are as: (1) Being kept from achieving “positively valued goals”; (2) Being deprived of “positively valued stimuli” and (3) Being presented with “noxious or negatively valued stimuli” [18]. In the context of the hurricane, at least the first two types of stain are relevant to displaced students as we are going to argue below. For the first type of strain, the hurricane forced affected students to interrupt their education for some period of time as it was found that on average, uprooted students had been away from school for nearly one month [2]. For the second type, one study enumerated a long list of life-events in the case of adolescents, such as breakup from boy/girl friend, death/illness of a friend, shifting to a new school district, divorce/separation of parents, and suspension from school [29]. It implies that those who left their schools, friends and even families had experienced much stress. Even worse, their homes might be destroyed by the hurricane and research showed that their family income underwent prolonged decrease post Katrina [3,4]. Several other studies confirmed the strain and related psychological/mental problems among displaced students [12,13,14,15,16]. According to GST, we thus expect that relative to non-evacuees, evacuee students would be more likely to be involved in in-school discipline infractions post Katrina as they had experienced the strain.

Another related theory is Travis Hirschi’s social control theory. According to this theory, crime/delinquency is intrinsically determined by human nature and “we are all animals and thus naturally capable of committing criminal acts” [30]. Thus, it is conformity instead of human nature that should be used to explain delinquency. Hirschi interprets conformity as “the formation of a bond between individual and society comprised of four major elements: attachment, commitment, involvement and belief” [31,32]. The stronger the social bond is, the less possibly the individual will commit any crime. When the bond is weak, people tend to depend on themselves and disregard rules of conduct, leading to delinquent behavior out of self interest [30]. Empirical research gave support to social control theory since its development [33,34,35]. Some studies tested the relevance of the theory in the context of natural disasters [36]. Thus, based on social control theory, we can predict that post Hurricane Katrina displaced students would be more likely to be engaged in delinquent behavior than non-evacuees due to the disturbance of social bond during the disaster and informal social control in new surroundings. 

In sum, in line with GST and social control theory, we have the following hypothesis regarding the displaced students’ behavior post Katrina:

***Hypothesis*** ***1:*** Relative to non-evacuees, displaced students would be more likely to be involved in in-school discipline infractions post Katrina.

## 2. Materials and Methods

### 2.1. Review of DID Analysis

**Figure 1:**
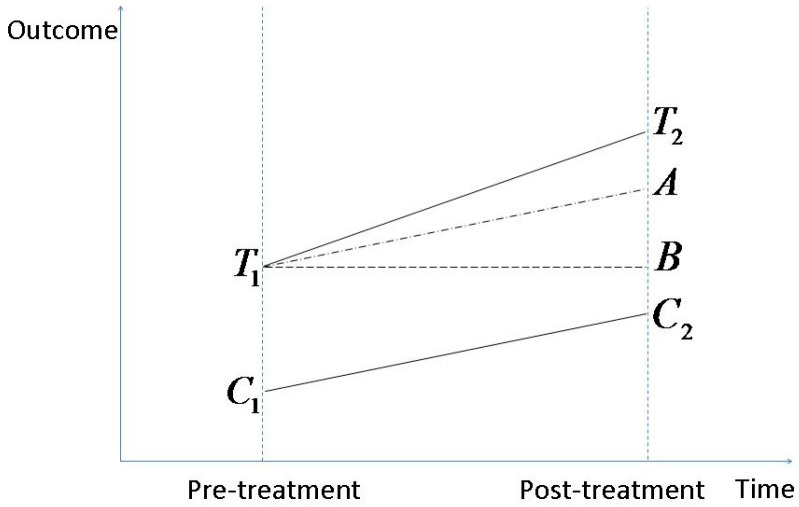
Graphic illustration of DID analysis.

DID analysis is a statistical method widely used in economics and quantitative sociology. It needs the data to be grouped in two groups (treatment *vs*. control group) and measured at two or more periods (pre- *vs*. post-treatment). Its purpose is to capture the effect of a treatment on an outcome by comparing the average over-time change of the outcome variable in the treatment group to the change in the control group. Figure 1 illustrates how DID analysis works. Line ${\text{T}}_{\text{1}}{\text{T}}_{2}$ represents the evolving of average outcome for the treatment group while ${\text{C}}_{1}{\text{C}}_{2}$ is for the control group. ${\text{T}}_{2}\text{B}$ measures the average change of the outcome variable for the treatment group pre and post the treatment, whereas AB equals the average over-time change for the control group. Thus, ${\text{T}}_{2}\text{A}$ stands for the effect of the treatment on the outcome variable.

Formally, DID analysis can be made by running the following regression:

(1) $$y_{\text{it}}=\alpha_{0}+\alpha_{1}T_{i}+\alpha_{2}P_{t}+\alpha_{3}(T_{i}\times P_{t})+\varepsilon_{it}$$

In Equation (1), subscripts i and t stand for individual and time period, respectively. ${\text{y}}_{\text{it}}$ represents the outcome variable; $T_{i}$ is a dummy indicating if treated or not. $P_{t}$ is a dummy equaling 1 if the time period is post treatment and 0 otherwise. $\varepsilon_{it}$ is the error term with conditional mean of zero. It is calculated that the change of the mean of y pre and post the treatment for the treatment group is $\alpha_{2}+\alpha_{3}$, whereas the over-time change for the control group is $\alpha_{2}$. The difference between the two coefficients is $\alpha_{3}$ and it is the coefficient of the interaction term, ($T_{i}\times P_{t}$), which captures the causal effect of the treatment on the outcome variable.

This paper explores the impact of Hurricane Katrina on students’ in-school behavior. Outcome variables are students’ discipline records at school, and the treatment is the displacement caused by Hurricane Katrina. The treatment group is Louisiana public school students who were ever displaced by Katrina in 2005 while the control group is other public school students in Louisiana observed in 2006. The pre-treatment time period is year 2004 and only one post-treatment period, 2006, is considered in the estimation.

One validity condition for DID analysis is the exogeneity of the treatment [17]. The natural-experiment attribute of the hurricane makes us confident that the treatment is quite exogenous. There is a concern, however, that students’ class peers may have influence upon their behavior (peer effects) whereas the treatment ($T_{i}$) is possibly correlated with the composition of their peers (omitted variable in the error term of Equation (1)). The problem of endogeneity will lead to biased estimation in the DID analysis. Further if we look after 2005, there would be more concern of endogeneity, as a large part of displaced students gradually returned to their original parishes and even schools [11].

In 2006, the first year after the hurricane, the potential endogeneity is of least concern, since immediately after Katrina, displaced students were hard to self-select their schools and peers. One related research stated that: “In fact, many evacuees were evacuated on buses without knowing where they were going. Others were able to drive but had very limited options in terms of where to go …” [10]. We thus choose year 2006 as the only post-treatment period in the analysis due to endogeneity issues.

Another key assumption behind the identification of DID analysis is that the treated and control groups should have *the same trend* in the outcome, and only in this case can the length of ${\text{T}}_{2}\text{A}$ in Figure 1 can be reasonably justified as the treatment effect of interest. We will test this parallel trend assumption before formally proceeding to the DID analysis below.

### 2.2. Data and Measures

#### 2.2.1. Data

The Louisiana Department of Education in the U.S. provided us the database of all Louisiana K12 students’ education records during 2000–2008. It includes students’ demographic information, grade, school, academic achievement, as well as family economic status, indicated by their eligibility for free lunch at school. More importantly, the database has information about the type and number of discipline infractions at school each academic year. The main purpose of this paper is to investigate the change of students’ likelihood for discipline infractions pre and post Hurricane Katrina. One concern about the data is its attrition after 2005 when Katrina happened. We observe records of 731, 351 students in 2004 while the number in 2006 is 681,753 with a loss of 6.78% observations. The attrition is due to many displaced students’ moving out of state. To see the difference between the students who stayed and those who disappeared from the data in 2006, we look at 2004 records and create a dummy indicating if the students disappeared in 2006. Then, we regress this dummy on a set of grade, school, economic status as well as demographic dummies. We find that male and black students as well as students at economic disadvantage (those eligible for free lunch) are more likely to disappear from the data (t-statistics of 2.05 for male, 2.28 for black and 2.44 for free lunch).

#### 2.2.2. The Dependent Variable

The Louisiana Department of Education database has information about the type and number of students’ discipline infractions at school each academic year. Table 1 lists the code of discipline records and its explanation. We categorize them into four types, *i.e*., status offense (with Code 01, 02, 04, 05, 08, 09, 10, 12, 13, 14, 15, 17, 18, 19, 31, 33 and 34), offense against person (with Code 03, 06, 16 and 35), offense against property (with Code 11 and 20) and serious crime (with Code 07, 21, 22, 23, 24, 25, 26, 27, 28, 29, 30 and 32). Then dummy variables are constructed reflecting if students are involved in any (each type of) discipline infractions each year.

**Table 1:** Discipline Code and its Explanation.

Offense	Code	Discipline Infraction
Status offense	01	Willful disobedience
-	02	Treats an authority with disrespect
-	04	Uses profane and/or obscene language
-	05	Is guilty of immoral or vicious practices
-	08	Uses or possesses tobacco or lighter
-	09	Uses or possesses alcoholic beverages
-	10	Disturbs the school or habitually violates any rule
-	12	Writes profane and/or obscene languages or draws obscene pictures
-	13	Possess weapons as defined in section 921 or title 18 or the US code
-	14	Possesses firearms, knives, or other implements, which can be used as weapons
-	15	Throws missiles liable to injure others
-	17	Violates traffic and safety regulations
-	18	Leaves school premises or classroom without permission
-	19	Is habitually tardy or absent
-	31	Possesses pocket knife with a blade length of less than 2.5 inches
-	33	Use of medication in a manner other than prescribed
-	34	Possession of body armor
Offense against person	03	Makes an unfounded charge against authority
-	06	Is guilty of conduct or habits injurious to his/her associates
-	16	Instigates or participates in fights while under school supervision
-	35	Bullying
Offense against property	11	Cuts, defaces, or injures any part of public school building
-	20	Is guilty of stealing
Serious crime	07	Uses or possesses any controlled dangerous substances governed by law
-	21	Commits any other serious offense
-	22	Murder
-	23	Assault and/or battery
-	24	Rape and sexual battery
-	25	Kidnapping
-	26	Arson
-	27	Criminal damage to property
-	28	Burglary
-	29	Misappropriation with violence to the person
-	30	Discharge or use of weapons prohibited by federal law
-	32	Serious bodily injury

Notes: This table is from data dictionary of Louisiana education database. Status offense refers to discipline infractions with code 01, 02, 04, 05, 08, 09, 10, 12, 13, 14, 15, 17, 18, 19, 31, 33 and 34; offense against person with code 03, 06, 16 and 35; offense against property with code 11 and 20; and serious crime with code 07, 21, 22, 23, 24, 25, 26, 27, 28, 29, 30 and 32.

#### 2.2.3. Independent Variables

In 2006, all Louisiana public schools assigned students a label of Katrina evacuee when their schools were closed, or their families were forced to move during the storm or both. Most displaced students from the most affected regions (e.g., Orleans Parish) ended up with a different school in the beginning of 2006 whereas many evacuees from less affected regions went back to their original schools when the hurricane was over. The label of evacuee was assigned no matter if displaced students returned or not and how long they had been away from original schools if they returned. We identify treatment group in this study according to this label and construct a dummy variable “Katr” which equals to “1” across all years when the student was assigned the label of evacuee in 2006 and 0 otherwise. 

Following the above equation [1], we also create a dummy “Post” which equals to 1 when the year is after 2005—the year of Hurricane Katrina and 0 otherwise. This dummy variable and its interaction term with “Katr”—“Katr*Post” are all included as independent variables. Other control variables include students’ demographic information (Male, Black, Hispanic and Asian), economic status (if eligible for free lunch at school) and a set of dummies for grade and school sites.

Table 2 reports descriptive statistics of main variables for the sample in 2006. There are 681,655 student records observed on that year. About 10.9 percent of them were displaced by Hurricane Katrina in 2005. More than half of students were eligible for free lunch at school, and black students consist of 44.6 percent of the sample. In 2006, around 23 percent of students were involved in discipline infractions and recorded by school. Status offense is the most common infraction whereas serious crime is least likely to happen. 

**Table 2:** Descriptive Statistics of Sample.

Variable	Mean (%)	SD (%)
Katr	10.9	32.4
Eligible for free lunch	56.1	49.7
Male	51.8	48.7
Black	44.6	47.6
Hispanic	1.9	13.1
Asian	1.7	12.6
Dummy for any infraction	23.3	42.5
Dummy for status offense	19.8	40.9
Dummy for offense against person	11.1	31.2
Dummy for offense against property	12.1	35.2
Dummy for serious crime	3.8	22.3

Notes: This table shows the descriptive statistics of the sample in 2006. Katr is a dummy variable indicating if students were evacuees displaced by Hurricane Katrina. Status offense refers to discipline infractions in Table 1 with code 01, 02, 04, 05, 08, 09, 10, 12, 13, 14, 15, 17, 18, 19, 31, 33 and 34; offense against person with code 03, 06, 16 and 35; offense against property with code 11 and 20; and serious crime with code 07, 21, 22, 23, 24, 25, 26, 27, 28, 29, 30 and 32.

### 2.3. Method of Analysis

#### 2.3.1. Preliminary Analysis

Before moving to difference-in-difference analysis, it is advisable to consider the evolving of displaced students’ discipline records over time relative to non-evacuees to see if there was a sudden change in evacuees’ behavior around 2005. For this purpose, we regress the following model year by year during 2001–2008 except 2005:

(2) $$Y_{i}=\alpha +\beta Katr_{i}+\gamma_{1}G_{i}+\gamma_{2}S_{i}+\gamma_{3}D_{i}+\gamma_{4}I_{i,-1}+\varepsilon_{i}$$

In Equation (2), $Y_{i}$ represents a dummy variable indicating if students were recorded of any discipline infractions. $Katr_{i}$ is the dummy for evacuee status. $G_{i}$ includes a set of grade dummies (the data have twelve grades; accordingly, we create dummies for grade 1–11 (grade 12 is used as the reference grade). The dummy for the 1st grade is assigned 1 if the student is of grade 1 and 0 otherwise. Other grade dummies are constructed similarly. For school dummies, the data provide the information of school district and school name. The dummy for some schools is assigned 1 if the students are from that school and 0 otherwise. Other school dummies are constructed similarly. $S_{i}$ is a vector including school dummies. In literature, many researchers discussed the effects of different educational context and teachers’ quality on students’ academic performance [37,38]. It would be reasonable to think that the school effects are working as teachers’ quality, teacher-student ratio, and the communities where schools are located can influence educational performance and may have effects on students’ discipline infraction. In addition, different schools may have different tolerance toward deviant behavior. Thus, we include school dummies in the analysis. $D_{i}$ contains a set of demographic dummy variables and $I_{i,-1}$ represents students’ one-period-lagged economic status. Economic status is lagged by one period to prevent potential endogeneity issues. For example, according to the general strain theory in criminology, students who were more affected by the hurricane would have experienced more strain and thus more discipline problems. Meanwhile, studies showed that more affected families during Katrina had experienced more income loss [3,4]. In this case, when current economic status is included in the right-hand side of Equation (2), it will be correlated with the error term. This endogeneity problem might bias the estimation. Hurricane Katrina did not randomly hit Louisiana. The coastal areas such as New Orleans were the most affected areas where African Americans and people at economic disadvantage constituted a higher proportion of the population than other regions of Louisiana [11]. As a result, $Katr_{i}$ is correlated with some demographic and socioeconomic-status variables; leaving these control variables in the error term will cause the omitted-variable bias in estimation.

Equation (2) is regressed repeatedly using the ordinary least square (OLS) method for each year sample and estimated standard errors of coefficients are all clustered at school levels. In addition, considering the possible effects of data attrition on estimated coefficients and making results more comparable across years, only students whose records exist in 2006 are used in the analysis. As the dependent variable is binary, Equation (2) is a linear probability model whose advantage over Probit/Logit model is that its coefficients can be interpreted easily [39]. After estimation, the coefficient *β* captures the likelihood of discipline infraction for the treatment group (evacuees) relative to the control group (non-evacuees) of that year. We look at the evolving of *β* across years, especially the change around 2005 to see the effect of the hurricane on displaced students’ behavior.

We will not estimate the above model for year 2005 considering that displaced students on average missed as many as five weeks’ class when Hurricane Katrina did happen [2]. Thus with less time at school, their in-school discipline records will not be comparable to that of non-evacuees in 2005.

#### 2.3.2. DID Analysis

We are going to investigate the effect of Hurricane Katrina on students’ in-school behavior in a difference-in-difference framework using the following linear probability model:

(3) $$Y_{it}=\alpha +\pi Katr_{i}+\theta Post_{t}+\delta Katr_{i}*Post_{t}+\tau_{1}G_{it}+\tau_{2}S_{it}+\tau_{3}D_{i}+\tau_{4}I_{i,t-1}+\varepsilon_{it}$$

In Equation (3), $Y_{it}$ represents dummies of any discipline infraction/status offense/offense against person/offense against property/serious crime, respectively, for student i at period t. $Post_{t}$ is a dummy variable indicating if the period is post 2005. As discussed above, students’ economic status is lagged by one period to prevent potential endogeneity issues.

In this DID specification, the treatment group is students displaced by the hurricane, indicated by the dummy variable - $Katr_{i}$. We have year 2004 as the pre-treatment period, and 2006 as the post-treatment period. As discussed above, including 2006 as the only post-treatment period is to prevent possible endogeneity problems. Considering the sample attrition during 2004–2006, we include only students whose records are observed in both 2004 and 2006 in our analysis (We also tried to include all samples of 2004 and 2006 in the analysis and found that the results are not qualitatively different). The coefficient of the interaction term—δ, captures the effect of the hurricane on students’ in-school behavior in this DID framework. Mathematically, it measures the change of displaced students’ likelihood of discipline infraction pre and post the hurricane relative to the change of non-evacuees as discussed before.

It is worth noting that we specify the DID analysis using a linear probability model instead of Probit or Logit model. This is because DID analysis using non-linear models (such as Probit/Logit) is generally complicated and the results are difficult to explain [40,41].

## 3. Results

### 3.1. Preliminary Analysis

The regressing results of Equation (2) are presented in Table 3. It is observed that before 2004, displaced students’ discipline behavior is not significantly different from others, while post Katrina, their likelihood of infraction is significantly higher and declining over the time. Other estimated coefficients are quite stable across the years. On average, male students’ probability of infraction is 0.124 higher than female; the probability for students eligible for free-lunch is 0.095 higher; and relative to white, black students’ probability is 0.13 higher with Asian 0.091 lower and Hispanic 0.026 lower.

**Table 3:** Displaced students’ relative likelihood of infraction across years.

	2001	2002	2003	2004	2006	2007	2008
Katr	−0.008	−0.0064	−0.0086	−0.0055	0.0654 ***	0.0234 ***	0.0124 ***
	(0.007)	(0.007)	(0.005)	(0.004)	(0.008)	(0.004)	(0.003)
Male	0.124 ***	0.122 ***	0.126 ***	0.125 ***	0.123 ***	0.127 ***	0.121 ***
	(0.002)	(0.002)	(0.002)	(0.002)	(0.002)	(0.002)	(0.002)
Black	0.12 ***	0.12 ***	0.12 ***	0.135 ***	0.14 ***	0.14 ***	0.136 ***
	(0.003)	(0.003)	(0.003)	(0.003)	(0.003)	(0.003)	(0.003)
Hispanic	−0.017	−0.033 ***	−0.029 ***	−0.031 ***	−0.028 ***	−0.02 **	−0.02 ***
	(0.009)	(0.008)	(0.007)	(0.007)	(0.007)	(0.007)	(0.006)
Asian	−0.078 ***	−0.1 ***	−0.087 ***	−0.1 ***	−0.095 ***	−0.1 ***	−0.091 ***
	(0.007)	(0.007)	(0.007)	(0.007)	(0.008)	(0.006)	(0.006)
If eligible for	0.09 ***	0.09 ***	0.09 ***	0.098 ***	0.1 ***	0.1 ***	0.1 ***
free lunch	(0.002)	(0.002)	(0.002)	(0.002)	(0.002)	(0.002)	(0.002)
R-square	0.16	0.16	0.16	0.16	0.18	0.18	0.18

Notes: Equation (2) is repeatedly regressed for each year except 2005, and only students whose records are on file in 2006 are included in the analysis. The dependent variable is a dummy indicating if students are involved in any discipline infraction. Katr is a dummy variable indicating if students were evacuees displaced by Hurricane Katrina. We estimate the linear probability model using OLS method. Standard errors clustered at school levels are in parenthesis. *, **, and *** mean 0.01, 0.005 and 0.001 significance level, respectively. Other estimated coefficients are not reported.

**Figure 2:**
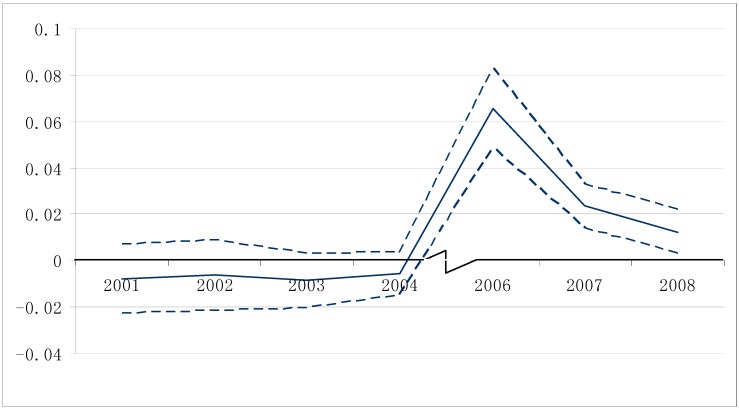
The evolving of evacuees’ likelihood of discipline infraction relative to non-evacuees. *Notes:* Equation (2) is regressed each year during 2001–2008 except 2005 when Hurricane Katrina happened. Only students whose records exist in 2006 are used in the analysis. The solid line connects estimated β of each year whereas the dashed lines form 95% confidence interval of each estimated β.

Figure 2 shows the changing of the likelihood of discipline infraction for displaced students relative to non-evacuees. The solid line connects estimated β of Equation (2) for each year and two dashed lines form 95% confidence intervals for each estimated β. Before 2004, displaced students’ likelihood of infraction is not statistically different from non-evacuees. However, there is a sharp increase in the likelihood for evacuees in 2006, indicating that Hurricane Katrina happening in 2005 worsens displaced students’ in-school behavior. The effect is declining afterwards but still remains statistically positive in 2007 and 2008.

We also do the analysis when $Y_{i}$ (in Equation (2)) represents dummies for status offense/offense against person/offense against property/serious crime, respectively. The results produce figures similar to Figure 2.

Figure 2 also reveals that for each pre-Katrina period, the estimated β of Equation (2) is not significantly different from zero, implying parallel trends of evacuee and non-evacuee students in the probability of discipline infraction before the hurricane. More formally, we further examine the parallel trend assumption using the falsification test as below.

#### 3.1.1. Falsification Test

To check if the treated and control groups have similar trends in discipline infraction, we regress Equation (3) using the data of two pre-Katrina periods 2002 and 2004. In this case, $Post_{t}$ is a dummy for the year 2004. Table 4 lists the results of this falsification test, showing that in all cases, the coefficients of the interaction term have flipping signs and are statistically insignificant. This indicates that during 2002–2004, the behavioral change of the students to be displaced by the hurricane one year later is not statistically different from others. We also make use of the data of other pre-Katrina years to do the falsification test and it yields similar results as reported in Table 4. Having shown that the parallel trend assumption is not violated in our data, we proceeded to the DID analysis. 

#### 3.1.2. DID Analysis

The main results are displayed in Table 5, when we regress Equation (3) using OLS method. Statistically significant coefficients are showed with starts while standard errors clustered at school levels are in parentheses below the coefficients. For each type of discipline infraction, the coefficient of the interaction term is positive and statistically significant, implying that relative to non-evacuees, displaced students’ in-school behavior worsened post Hurricane Katrina. Specifically, displaced students’ likelihood of any discipline infraction increased relatively by 7.3% due to the hurricane. And the increase in the likelihood for status offense, offense against person, offense against property and serious crime is 4%, 1.5%, 3.8% and 2.1%, respectively. Note that these effects are sizeable compared to the incidences of respective infractions in the sample. For example, according to Table 2, in 2006, the incidence of any discipline infraction is 23.3%; thus the ratio of the relative increase of displaced students in their likelihood of any infraction (7.3%) to its incidence in the sample (23.3%) is as high as 31.3%. The ratio for status offense/offense against person/offense against property/serious crime is 20.2%, 13.5%, 31.4% and 55.2%, respectively. On the other hand, the effect of Hurricane Katrina on evacuees’ behavior seems to be short-termed although sizeable. As their hurricane-related strain is gradually relieved and social bond strengthens, the relative deviancy of displaced students declined over time, as shown in Figure 2. 

**Table 4:** Falsification Test for Parallel Trend Assumption (N = 1,352,702).

Independent Variable	Dependent Variable				
	Dummy for Any Infraction	Dummy for Status Offense	Dummy for Offense against Person	Dummy for Offense against Property	Dummy for Serious Crime
Katr	0.0035	−0.0044	0.0033	−0.0048	0.0021
	(0.0064)	(0.0071)	(0.0045)	(0.0056)	(0.0039)
Katr*Post	0.093	−0.140	−0.0615	0.089	0.021
	(0.149)	(0.214)	(0.093)	(0.144)	(0.225)
Post	−1.25	0.25	−1.58	−0.33	0.75
	(99.6)	(45.4)	(21.3)	(122.5)	(65.2)
**R-square**	0.11	0.15	0.021	0.057	0.033

Notes: Equation (3) is regressed using the sample of 2002 and 2004. Only students whose records were on file in both 2002 and 2004 are included in the analysis. Katr is a dummy variable indicating if students were evacuees displaced by Hurricane Katrina. We estimate the linear probability model using OLS method. Standard errors clustered at school levels are in parenthesis. Status offense refers to discipline infractions in Table 1 with code 01, 02, 04, 05, 08, 09, 10, 12, 13, 14, 15, 17, 18, 19, 31, 33 and 34; offense against person with code 03, 06, 16 and 35; offense against property with code 11 and 20; and serious crime with code 07, 21, 22, 23, 24, 25, 26, 27, 28, 29, 30 and 32. Other estimated coefficients are not reported.

We also find that male and black students are more likely to breach rules while Hispanic and Asian students are less likely to do that. Students whose economic status is at disadvantage are also more likely to be involved in discipline infractions.

In addition, it is interesting to note that the discipline infraction does not distribute uniformly across different grades. For each type of infraction, students from the 12th grade are most likely to breach the rule, resulting in negative coefficients for other grade dummies. Usually, students’ discipline records deteriorate as they mature (thus with higher grades). It seems to be a jump in probability of infraction at the sixth grade. So, middle- and high-school students (grades 6–12) have much higher deviancy rates. To check further if the relative increase in discipline infraction also amplifies among that group, we regress Equation (3) again using the sub-sample of students with grade 6–12. Table 6 shows that middle- and high-school students have higher increase in the risk of any breach. Regarding the subcategories, their relative risk increment is also higher in the status and property offense. Though, their likelihood of offense against person and serious crime undergoes similar over-time change as other groups. 

**Table 5:** Effect of Hurricane Katrina on displaced students’ likelihood of discipline infraction (N = 1,136,250).

Independent Variable	Dependent Variable				
	Dummy for Any Infraction	Dummy for Status Offense	Dummy for Offense against Person	Dummy for Offense against Property	Dummy for Serious Crime
Katr	−0.0079	−0.0085	−0.003	−0.0049	0.0017
	(0.0096)	(0.0098)	(0.0024)	(0.0057)	(0.003)
Katr*Post	0.073 ***	0.040 ***	0.015 ***	0.038 ***	0.021 ***
	**(0.0049)**	**(0.0045)**	**(0.003)**	**(0.0041)**	**(0.0035)**
Post	1.08	−0.61	−0.132	0.39	−0.18
	(111.6)	(33.2)	(35.4)	(21)	(36.3)
**Male**	0.121 ***	0.166 ***	0.048 ***	0.07 ***	0.036 ***
	(0.0009)	(0.0019)	(0.0005)	(0.0009)	(0.0006)
**Black**	0.14 ***	0.232 ***	0.022 ***	0.073 ***	0.033 ***
	(0.0012)	(0.002)	(0.0007)	(0.001)	(0.0009)
**Hispanic**	−0.025 ***	−0.027 ***	−0.015 ***	−0.025 ***	−0.002
	(0.003)	(0.005)	(0.001)	(0.0029)	(0.0027)
**Asian**	−0.094 ***	−0.074 ***	−0.018 ***	−0.041 ***	−0.032 ***
	(0.003)	(0.004)	(0.0014)	(0.0026)	(0.0019)
**If eligible for**	0.11 ***	0.067 ***	0.025 ***	0.051 ***	0.019 ***
**free lunch**	(0.0012)	(0.0009)	(0.0006)	(0.0008)	(0.0007)
**1st grade**	−0.453 ***	−0.231 ***	−0.194 ***	−0.106 ***	−0.094 ***
	(0.004)	(0.004)	(0.004)	(0.003)	(0.003)
**2nd grade**	−0.427 ***	−0.216 ***	−0.176 ***	−0.102 ***	−0.091 ***
	(0.004)	(0.004)	(0.004)	(0.003)	(0.003)
**3rd grade**	−0.403 ***	−0.204 ***	−0.162 ***	−0.097 ***	−0.088 ***
	(0.004)	(0.004)	(0.004)	(0.003)	(0.003)
**4th grade**	−0.354***	−0.176***	−0.136***	−0.088***	−0.08***
	(0.004)	(0.004)	(0.004)	(0.003)	(0.003)
**5th grade**	−0.314 ***	−0.15 ***	−0.117 ***	−0.079 ***	−0.076 ***
	(0.004)	(0.004)	(0.004)	(0.003)	(0.003)
**6th grade**	−0.212 ***	−0.088 ***	−0.066 ***	−0.031 ***	−0.05 ***
	(0.004)	(0.04)	(0.004)	(0.003)	(0.003)
**7th grade**	−0.171 ***	−0.057 ***	−0.058 ***	−0.016 ***	−0.038 ***
	(0.004)	(0.005)	(0.004)	(0.003)	(0.003)
**8th grade**	−0.185 ***	−0.06 ***	−0.074 ***	−0.032 ***	−0.043 ***
	(0.004)	(0.004)	(0.004)	(0.003)	(0.003)
**9th grade**	−0.166 ***	−0.048 ***	−0.059 ***	−0.039 ***	−0.043 ***
	(0.004)	(0.004)	(0.004)	(0.003)	(0.003)
**10th grade**	−0.175 ***	−0.065 ***	−0.062 ***	−0.044 ***	−0.046 ***
	(0.004)	(0.005)	(0.004)	(0.003)	(0.003)
**11th grade**	−0.216 ***	−0.096 ***	−0.076 ***	−0.06 ***	−0.053 ***
	(0.005)	(0.005)	(0.005)	(0.003)	(0.003)
**R-square**	0.18	0.13	0.067	0.076	0.064

*Notes*: Equation (3) is regressed using the sample of 2004 and 2006. Only students whose records were on file in both 2004 and 2006 are included in the analysis. Katr is a dummy variable indicating if students were evacuees displaced by Hurricane Katrina. We estimate the linear probability model using OLS method. Standard errors clustered at school levels are in parenthesis. *, **, and *** mean 0.01, 0.005 and 0.001 significance level, respectively. Status offense refers to discipline infractions in Table 1 with code 01, 02, 04, 05, 08, 09, 10, 12, 13, 14, 15, 17, 18, 19, 31, 33 and 34; offense against person with code 03, 06, 16 and 35; offense against property with code 11 and 20; and serious crime with code 07, 21, 22, 23, 24, 25, 26, 27, 28, 29, 30 and 32.

**Table 6:** Effect of Hurricane Katrina on displaced students’ likelihood of discipline infraction (Only students with grade 6–12 are used in the analysis, N = 617,896).

Independent Variable	Dependent Variable				
	Dummy for any Infraction	Dummy for Status Offense	Dummy for Offense against Person	Dummy for Offense against Property	Dummy for Serious Crime
Katr	−0.0016	−0.0047	−0.0031	−0.0021	−0.003
	(0.0707)	(0.0064)	(0.0045)	(0.0053)	(0.005)
Katr*Post	0.087 ***	0.057 ***	0.012 ***	0.049 ***	0.025 ***
	**(0.003)**	**(0.002)**	**(0.002)**	**(0.003)**	**(0.003)**
Post	0.98	0.54	1.15	−0.41	−1.65
	(31.5)	(21.4)	(62.3)	(19.6)	(25.7)
**R-square**	0.16	0.15	0.05	0.091	0.055

Notes: Equation (3) is regressed using the sample of 2004 and 2006. Only students of grade 6–12 whose records were on file in both 2004 and 2006 are used in the analysis. Katr is a dummy variable indicating if students were evacuees displaced by Hurricane Katrina. We estimate the linear probability model using OLS method. Standard errors clustered at school levels are in parenthesis. *, **, and *** mean 0.01, 0.005 and 0.001 significance level, respectively. Status offense refers to discipline infractions in Table 1 with code 01, 02, 04, 05, 08, 09, 10, 12, 13, 14, 15, 17, 18, 19, 31, 33 and 34; offense against person with code 03, 06, 16 and 35; offense against property with code 11 and 20; and serious crime with code 07, 21, 22, 23, 24, 25, 26, 27, 28, 29, 30 and 32. Other estimated coefficients are not reported.

## 4. Discussion

One feature of the data used in this study is that many affected students disappeared from the sample post the hurricane when they moved to other states. As we showed before, the attrition is far from random which makes it difficult to identify the causal effects of the disaster on students’ behavior in the following ways. One point is that those who are most deprived will be more likely to stay, as traveling needs some least amount of resources. As a consequence, what constitutes the sample in our DID analysis will be over-represented by students from more deprived families who are subject to more strain and expected to conduct more deviant behavior, according to Agnew’s GST. In this case, the results reported in Table 5 will be upward biased. It is also possible that the most deprived families will be ready to move as they have less to give up in Louisiana. Put another way, their opportunity cost of moving is less. Then most data would consist of less deprived families’ children and thus estimated results would be downward biased. The second hypothesis seems to be more relevant to our data as around 78.7 percent of evacuees were eligible for free lunch in 2004 while the number for those who had disappeared post Katrina is as high as 93.5 percent. Since families at economic disadvantage are more vulnerable (e.g., with shelters of lower quality and less means to cope with the disaster, *etc*.) and prone to be more deprived during the storm. It would be reasonable to infer that the displaced students moving out of state were more likely to be from the most deprived families. 

A related study estimated that the students who disappeared from the data did not originate from each parish with equal likelihood [11]. Students from Orleans Parish (the most affected parish) have 0.308 higher disappearance probability than other parishes. Also considering that around 35.9 percent of evacuees come from that parish, we decide to re-do the DID analysis excluding the samples originating from Orleans Parish. In this case, the coefficient identification would be less affected by the data attrition.

Table 7 reports the main results of our analysis when excluding Orleans Parish-originating students from the data. The relative increase of displaced students in the likelihood of overall infraction/offense against person/serious crime is slightly lower while the increase in status offense/offense against property is higher than the Table 5. But generally, the results in these two tables are quite similar. Although the results in Table 5 are likely to be subject to less bias due to data attrition, the sample without students from Orleans Parish invites another bias: because Orleans Parish is one of the most affected parishes during the hurricane and students there are accordingly more likely to be the most deprived ones, their strain is supposed to be greater as well. Then in line with Agnew’s GST, they are expected to conduct more deviancy than students from other regions. Leaving them out of sample will result in a downward bias for the results in Table 7. This study cannot fully partial out the role of data attrition in the results because of the data availability issue; it is left for future research when the data on evacuee students in Texas and other states are accessible.

**Table 7:** Effect of Hurricane Katrina on displaced students’ likelihood of discipline infraction (students originating from Orleans Parish excluded, N = 987,248).

Independent Variable	Dependent Variable				
	Dummy for Any Infraction	Dummy for Status Offense	Dummy for Offense against Person	Dummy for Offense against Property	Dummy for Serious Crime
Katr	−0.0087	−0.0056	0.0045	−0.0091	0.0019
	(0.0107)	(0.0085)	(0.0053)	(0.0121)	(0.005)
Katr*Post	0.069 ***	0.047 ***	0.011 ***	0.041 ***	0.018 ***
	**(0.0022)**	**(0.0034)**	**(0.002)**	**(0.0039)**	**(0.0024)**
Post	1.76	−0.81	−1.45	0.31	−1.33
	(121.2)	(63.4)	(35.4)	(78.4)	(75.5)
**R-square**	0.17	0.14	0.061	0.082	0.059

Notes: Equation (3) is regressed using the sample of 2004 and 2006. Only students whose records were on file in both 2004 and 2006 are included in the analysis. In addition, students who originate from Orleans Parish in 2004 are excluded from the data. Katr is a dummy variable indicating if students were evacuees displaced by Hurricane Katrina. We estimate the linear probability model using OLS method. Standard errors clustered at school levels are in parenthesis. *, **, and *** mean 0.01, 0.005 and 0.001 significance level, respectively. Status offense refers to discipline infractions in Table 1 with code 01, 02, 04, 05, 08, 09, 10, 12, 13, 14, 15, 17, 18, 19, 31, 33 and 34; offense against person with code 03, 06, 16 and 35; offense against property with code 11 and 20; and serious crime with code 07, 21, 22, 23, 24, 25, 26, 27, 28, 29, 30 and 32. Other estimated coefficients are not reported.

This paper uses administrative education data instead of students’ self reports. One problem with this data is that students’ discipline records will be affected by schools’ threshold of reporting or tolerance toward discipline infraction, of which we have no information. It is conceivable that when transferring to a new school, the displaced students are less tolerated by their new teachers with whom they are unfamiliar and reported more infractions than native students. In this case, the estimated results may be upward biased. There is another possibility that displaced students receive more tolerance toward deviancy as teachers might have sympathy for the victims of the devastating disaster and understand that they are subject to greater strain, which, on the contrary, will result in downward biased estimators. However, we cannot directly test these hypotheses in this paper due to the lack of additional data. 

We compare the likelihood of infraction pre and post the hurricane in a linear probability model instead of comparing frequency of offense using count regression models because the different offenses within each type of infraction are not readily comparable. For example, suppose that a student was caught “Possessing firearms, knives or other implements, which can be used as weapons” once in 2004 whereas he/she was found “Using or possessing tobacco or lighter” and “Using or possessing alcoholic beverages” once for each in 2006. Can we conclude that he/she is more deviant in 2006 because of two status offenses compared to only one in 2004? Thus the limited comparability between offenses makes count models less attractive in the context of this study (However, the change of frequency of infraction pre and post the hurricane for some typical category of offense under each type of infraction using a negative binomial model was analysed, and can be found in the Appendix. The results also indicate significant relative increase in deviance for displaced students). 

Another concern is that the estimated results in this study might be due to the difference between pre- and post-Katrina schools if displaced students moved to schools with more crime or strain in general. This concern is partly alleviated as we have added school dummies in Equation (3) that captures the school effect on students’ deviancy (e.g., the effect of different teacher quality, tolerance toward infraction and crime rate of the communities where schools are located *etc*. on students’ discipline records). However, the school dummies will not capture the effects of different peers on evacuees’ behavior when they are transferred to new schools. In the case of Katrina students, a large portion of evacuees was from New Orleans whose K12 education was worse than the Louisiana average before the hurricane. As a result, beside disruption, Hurricane Katrina also offered them the opportunity of better schooling. A study showed that “in the medium run, the New Orleans evacuees have seen increased academic achievement as a result of being kicked out of their original schools” [11]. A natural expectation is that while moving to better schools, these evacuees would have better class peers than before (in another study, through interviewing the teachers in evacuees’ new schools, the authors find that displaced students are more deviant than their new peers “in terms of truancy, fighting and engaging in risky behaviors” [10]). Taking into account the positive peer effect on reducing evacuees’ deviancy, the estimated results in Table 5 are likely to be under-estimated.

Last but not least, this paper contributes to the literature of GST in that it is the first paper testing Agnew’s GST in the DID framework. Except studies using longitudinal data [27,42,43] or recently in the context of a randomized experiment [44], most research empirically testing GST makes use of cross-sectional survey data, leaving the problem of endogeneity unsolved. In this study, the exogeneity of evacuee status of students during the storm makes the identification least affected by the endogeneity issue.

## 5. Conclusions

This paper examines the effect of Hurricane Katrina on displaced students’ in-school behavior using a unique dataset. Difference-in-difference (DID) approach is used for the analysis. The results show that relative to non-evacuees, displaced students’ likelihood of discipline infraction increased by 7.3% after the hurricane. Thus, when disasters occur as was the case with Hurricane Katrina, in addition to assistance for adult evacuees, government in cooperation with schools should provide aid and assistance to displaced children to guarantee their mental health and in-school behavior.

## Appendix

Previously we argued that analyzing the change of frequency of deviance for each type of infraction is not attractive since different categories of infraction under the same type are not readily comparable. However, we can instead analyze the change of frequency count for each *category* of infraction using count models. There are 35 categories of offense in Table 1. We thus pick up only one category of offense for each type of infraction for this purpose. Considering that the data exhibits over-dispersion, *i.e*., the variance of deviance count is much greater than the mean, we use the following negative binomial instead of the Poisson model for the analysis:

(4) $$E(Y_{it}|X_{it})=\exp(\alpha +\theta Post_{t}+\pi Katr_{i}+\delta Katr_{i}*Post_{t}+\tau_{1}G_{it}+\tau_{2}S_{it}+\tau_{3}D_{i}+\tau_{4}I_{i,t-1})$$

where $Y_{it}$ stands for the number of infraction and $X_{it}$ for all control variables. Equation (4) will be estimated using the maximum likelihood method. The coefficient δ captures the relative increase of displaced students in deviance count (in logs) and is expected to be positive.

**Table A1:** Effect of Hurricane Katrina on displaced students’ expected number (in logs) of infraction (N = 1,136,250).

Independent Variable	Dependent Variable			
	Num of Willful Disobedience	Num of Participating in Fight	Num of Being Guilty of Stealing	Num of Assault/Battery
Katr	−0.086	−0.202	−0.048	0.133
	(0.095)	(0.311)	(0.065)	(0.254)
Katr*Post	**0.47 *****	**0.56 *****	**0.36 *****	**0.59 *****
	**(0.045)**	**(0.092)**	**(0.027)**	**(0.041)**
Post	0.081	0.09	−0.037	−0.133
	(0.102)	(0.102)	(0.069)	(0.171)
**Male**	0.797 ***	1.26 ***	0.584 ***	0.774 ***
	(0.007)	(0.014)	(0.007)	(0.012)
**Black**	0.907 ***	0.349 ***	0.842 ***	0.55 ***
	(0.009)	(0.015)	(0.009)	(0.015)
**Hispanic**	−0.191 ***	−0.54 ***	−0.221 ***	0.049
	(0.038)	(0.065)	(0.041)	(0.045)
**Asian**	−0.986 ***	−1.01 ***	−0.634 ***	−0.943 ***
	(0.066)	(0.104)	(0.054)	(0.086)
**If eligible for**	0.664 ***	0.608 ***	0.559 ***	0.526 ***
**free lunch**	(0.009)	(0.016)	(0.009)	(0.015)

Notes: Equation (4) is regressed using the sample of 2004 and 2006. Only students whose records were on file in both 2004 and 2006 are included in the analysis. Katr is a dummy variable indicating if students were evacuees displaced by Hurricane Katrina. We estimate the negative binomial model using the maximum likelihood method. Standard errors clustered at school levels are in parenthesis. *, **, and *** mean 0.01, 0.005 and 0.001 significance level, respectively. *Willful disobedience* is with code 01 in Table 1; *participating in fight* with code 16; *being guilty of stealing* with code 20; and *assault/battery* with code 23. As Table A1 shows, the coefficient of the interaction term is significantly positive in all cases, implying more deviance of displaced students post Hurricane Katrina relative to non-evacuees.
